# Supplementary material for: Development of an intervention to improve access to living-donor kidney transplantation (the ASK study)
Source: PLoS One. 2021 Jun 25;16(6):e0253667. doi: 10.1371/journal.pone.0253667 (PMC8232417; doi:10.1371/journal.pone.0253667)
Supplement: S1 Table — (DOCX) [file pone.0253667.s001.docx]

**S1 Table.** **Changes to intervention components and resources**

| **Resource/component: Invitation letter from healthcare practitioners to potential donors** | | | | | |
| --- | --- | --- | --- | --- | --- |
| **Positive comments** | **Negative comments** | **Possible change(s)** | **Agreed change?** | **Reason for change(s)** | **Priority**  **(MoSCoW)** |
| **Not a personal request by/from patient:** ‘It’s coming from a third party so in some ways … I would feel less selfish if that letter was being sent out even though I’m the person who says, this is a list of people’s addresses.’ (Patient/Male/70-79 years/University postgraduate degree/Retired)  **Short and focused:** ‘As an initial start I think it’s great because I think if it goes into too much about kidney swapping and all that palaver then… I think it’s short, to the point, but it’s also asking for help.’ (Patient/Male/60-69 years/Vocational or technical training/Unemployed)  **Not a direct request for donation:** ‘It’s a very generic type letter as much as it’s targeted for that patient’s friendship group of family group or whatever, but it’s still quite generic. It’s saying one of the ways that they could be treated is a transplant and you could help, it’s not saying ‘Will you?’ is it?’ (Nurse other/Female/40-49 years) | **1. Need for warning:** ‘I would expect them to say something like ‘my doctor has asked me to list every possible human I know who might be able to so you might get a letter, don’t feel any pressure.’’ (Family member/Female/30-39 years/University postgraduate degree/Part-time employment) | **1. Participants to inform family/friends to expect letter:** At meeting with HCP to discuss social network and individuals to whom letter will be sent, encourage participant to inform recipients a letter is being sent. Give example sentence ‘My hospital team have asked me to send some information about my kidney problems to family and friends in case they want to help. I hope that’s okay.’ | **Yes** | **1. Important for outcome** (likely to improve acceptability to letter recipients); **Consistent with guiding principles** (to encourage patient-led engagement with social support network); **Uncontroversial and easy** | **Should have** |
|  | **2. Written in English – risk of excluding individuals who do not read English:** ‘I think that’s a good idea as long as the people you’re sending it out to have got English as a first language and they’re literate. You can’t make assumptions.’ (Nurse other/Female/50-59 years) | **2. Translation of documents** | **No – not for feasibility study – if intervention acceptable and feasible, plan to do in main RCT – tailoring for individuals who don’t read or speak English likely to require further adaptation beyond simple translation.** | **2. Important for outcome** (in specific groups); **Consistent with guiding principles** (to increase LDKT knowledge amongst people with kidney disease and their social network); | **Could have** |
|  | **3. Any written information risks excluding individuals with poor literacy and health literacy:** ‘If literacy is a problem I think that would require more of a conversation with the patient and to find out – is there someone that can go through the letter with them? That kind of thing.’ (Nurse other/Female/50-59 years)  ‘I guess writing letters or sending information leaflets brings up the whole problem about health literacy again. Are people going to read it? Are they going to understand what’s been written? Are they going to ignore it?’ (Transplant physician or surgeon/Female/30-39 years) | **3. Ensure language simple and letter short** (Flesch-Kincaid score - plain English. Easily understood by 13- to 15-year-old students, Comparable to Harry Potter books)**, Combine with other components as planned (LDKT discussion with healthcare practitioner, animations, home visit)** | **Yes** | **3. Important for outcome; Consistent with Guiding Principles** (to increase LDKT knowledge amongst people with kidney disease and their social network, to tailor information to individuals with limited health literacy); **Uncontroversial and easy; Repeated by several participants** | **Must have** |
|  | **4. Expected response needs to be clear:** ‘Are they just telling me about it, or do they want me to do something?’ (Transplant nurse or coordinator/Female/50-59 years)  ‘Am I being asked to do this or am I being nominated to do this?’ (Patient/Male/40-49 years/White/University postgraduate degree/Full-time employment) | **4. Include sentences in letter making next steps clear** ‘You may want to know how you can help. Sometimes family members and friends want to give one of their kidneys to the person who needs a kidney transplant. There is more information in the leaflet we have sent with this letter and via the video links below. If you would like to find out more, please get in touch with nurse Ann Example.’ | **Yes** | **4. Important for outcome** (likely to improve engagement); **Consistent with guiding principles** (to engage directly with an individual’s social support network); **Uncontroversial and easy; Repeated by several participants** | **Must have** |
|  | **5. Avoid personalisation/targeting an individual:** ‘I think for me that initial phase – the more kind of anonymous and official it seems then that’s better than it being ‘Dear…’ (Nurse other/Female/50-59 years) | **5. Remove personalised aspects of letter** i.e. Dear XXX and the insertion of the transplant candidate’s name. | **No – not consistent with guiding principles to engage directly with an individual’s social support network** |  |  |
| **Resource/component: Information leaflet on living kidney donation** | | | | | |
| **Good levels of detail:** ‘It explained everything in really good detail … it gives you all the details you need in a booklet.’ (Patient/Male/60-69 years/White/Vocational or technical training/Unemployed)  **Short/appropriate length:** ‘It’s not too long I don’t think because it tells you, as I say I think it’s an absolutely superb booklet, because it does tell you everything you need to know, just a bit too much in that middle bit.’ (Patient/Male/60-69 years/Vocational or technical training/Unemployed)  **Well-illustrated:** ‘I think this is great, yes. I mean the words are not – what you wanna avoid is having a huge chunks of big text. When it’s pretty broken up like this with informative pictures, it’s actually, yes. I mean it’s got a lot of information as well.’ (Patient/Male/30-39 years/Secondary school/Unemployed)  **Good to reference and return to:** ‘The thing about the little booklet is you can flip your way through it and then you can turn back a couple of pages just to check something and I think that still is – and then you can leave it on the side and come back to it and there is an advantage to having a small booklet.’ ((Patient/Male/70-79 years/University postgraduate degree/Retired)) | **1. Need for simple language:** ‘I’ve been on the ward and you can see their [patients’] faces, then I’ve translated what they’ve [Doctors] said. They’re on about they want you to drink more… don’t use these posh words, just say, ‘You need to drink more’. Because people don’t know the word bowels, and stuff like that, they don’t. Sometimes I’ve actually got to say, ‘Have you had a poo?’ it’s literally that, or, ‘Have you broke wind?’ because we’re asking them ‘Is it all working down there?’’ (Nurse other/Female/50-59 years) | **1. Use simple language in leaflet** e.g. replace urine with wee, replace cardiac with heart | **Yes** | **1. Important for outcome** (to increase acceptability and engagement)**; Consistent with guiding principles** (to tailor information to individuals with limited health literacy)**; Uncontroversial and easy; Repeated by several participants** | **Must have** |
|  | **2. Section entitled ‘What tests will I need to give a kidney?’ too long:** ‘that seems like a lot of stuff to take in. It mentions a psychologist, so I don’t know whether that’s just me or I think it’s a bit, it gives you all the information you need, but it may be a bit too much to read.’ (Patient/Male/60-69 years/Vocational or technical training/Unemployed) | **2. Reduce the section entitled ‘What tests will I need to give a kidney?’ – currently across 2 pages – reduced to 1 page** | **Yes** | **2. Important for outcome** (to increase acceptability and engagement)**; Consistent with guiding principles** (to tailor information to individuals with limited health literacy)**; Uncontroversial and easy;** | **Should have** |
|  | **3. Lack of personal stories/ accounts of donation and transplantation:**  ‘It comes across as more powerful if they hear a real life story because it’s something that they can relate to.’ (Patient/Male/30-39 years/Secondary school/Unemployed)  ‘I did make a note about [no] personal experiences in it. I think with the liver transplant I had quite a few leaflets, quite a few personal experiences in that I think if I remember rightly.’ (Patient/Male/60-69 years/Vocational or technical training/Unemployed) | **3. Add personal accounts of donation and transplantation** | **Yes** | **3. Important for outcome** (to increase acceptability, persuasiveness, and engagement)**; Consistent with guiding principles** (to increase LDKT knowledge amongst people with kidney disease and their social network)**; Uncontroversial and easy; Repeated by several participants** | **Must have** |
|  | **4. Statement that payment for donation is illegal seen as unnecessary:**  ‘I can’t imagine why they’d put ‘Will I get paid for donating a kidney?’ It shouldn’t even arise. Our system in England, sorry the UK, where we’ve given blood for decades, it’s never been paid for, you know, so it’s the altruistic nature is taken for granted I think so I’d leave that out personally.’ (Patient/Male/70-79 years/University postgraduate degree/Retired) | **4. Remove section on payment for donation being illegal** | **No – important to highlight legal boundaries on donation** |  |  |
| **Resource/component: Informational animations** | | | | | |
| **Accessible:** ‘I think it needs to be in this day and age it needs to be accessible for, albeit video or written down, so a wide range and nowadays podcasts and YouTube and all that sort of thing’ (Transplant nurse or coordinator/Female/50-59 years)  **Comprehensive content:** ‘Interviewer: What do you think about that?  Participant: Well it’s good news.  Interviewer: What would you change in that video?  Participant: Nothing. It’s all there really.’ (Patient/Female/40-49 years/Vocational or technical training/Unemployed)  **Simple, eye-catching:** ‘I thought the American little videos were – well they were simplistic weren’t they. Visually easy to assimilate. … I thought the content was enough to catch people’s attention, ask one or two questions.’ (Patient/Male/70-79 years/University postgraduate degree/Retired) | **1. Difficult to use as a reference:**  ‘the thing about those little videos is they run and then they’re gone and it’s a bit like, you know, the Kindle generation, it’s there but it’s not actually in your hand so you flick back and have a look.’ (Patient/Male/70-79 years/University postgraduate degree/Retired) | **1. Use in combination with written literature** | **Yes** | **3. Important for outcome** (to increase acceptability, persuasiveness, and engagement); **Consistent with guiding principles** (to increase LDKT knowledge amongst people with kidney disease and their social network); **Uncontroversial and easy;** | **Should have** |
|  | **2. Need to be tailored for UK:**  ‘I mean I’m not oversold on the American accent and of course the references to who would be paying and where you’re going to have it done would have to be changed for UK eyes.’ (Patient/Male/70-79 years/University postgraduate degree/Retired) | **2. i) Change voiceover from USA accent to English accent; ii) Replace maps of USA to UK; iii) Remove references to medical fees and insurance; iv) Change USA cultural references to UK references e.g. replace reference to dollar bill with reference to £10 note** | **Yes** | **2. Important for outcome** (to increase acceptability, persuasiveness, and engagement); **Consistent with guiding principles** (to increase LDKT knowledge amongst people with kidney disease and their social network); **Uncontroversial and easy;** | **Must have** |
| **Resource/component: Home visit content** | | | | | |
| **Empowering whole family:** ‘I quite like that idea, information is really powerful for the patient, and for their relatives. Whether or not they proceed to living kidney transplant or not, family and friends are going to be part of that person’s journey, through dialysis, transplantation, hospital admission, medication, all of that. So, even if it doesn’t necessarily end with a relative donating a kidney, I still think that is really valuable.’ (Transplant physician or surgeon/Female/30-39 years)  ‘I mean for her [mum – not suitable as a donor] to be better in the know only gonna be more beneficial for me and obviously anybody that’s close to me, if they know, if they’re better educated about my situation then that might make them feel confident enough to help me ‘cause they know what to do, they would know how to be effective in helping me.’ (Patient/Male/30-39 years/Secondary school/Unemployed)  **Education may be of direct benefit to relatives at risk of kidney disease:** ‘…if any of them went to go onto kidney trouble, they already have knowledge of it.’ (Transplant physician or surgeon/Male/40-49 years)  ‘Yeah, and potentially that prevents further kidney disease in the future for that group of people hopefully, if it’s a public health type programme. In the same way that we have anti-smoking and whatever, people could look after their blood pressure etc and then their kidneys will hopefully be better in the future.’ (Nurse other/Female/40-49 years) | **1. Content needs to be broad – not just on living donation:**  ‘I think you need to teach about everything, because they so need to know why kidney disease is so debilitating for people. That’s why they know, kidney failure, need a transplant, but people might not know everything else that’s gonna lead up to it. It's not about persuading them to it being a good idea, it's also helping them understand life is gonna be hard for that person, you might not want to give your kidney because you might have a young family, but actually you can at least understand what they are going through.’ (Home dialysis nurse/Male/30-39 years)  ‘I think it should cover a broader spectrum, definitely. Otherwise it does seem a bit like, ‘they’ve got that and what we want is this’. I think they respond better to a whole overview of things, so they know and they understand. People are quite good when they understand why kidney failure has happened.’ (Transplant nurse or coordinator/Female/50-59 years) | **1. Education session to cover kidney disease, dialysis, transplantation and living donation** | **Yes** | **1. Important for outcome** (to increase acceptability, persuasiveness, and engagement); **Consistent with guiding principles** (to increase LDKT knowledge amongst people with kidney disease and their social network, to increase an individual’s level of patient activation, to engage directly with an individual’s social support network, including potential donors)**; Uncontroversial and easy; Repeated by several participants** | **Must have** |
|  | **2. Tailored to individual**  ‘I think you have to totally individualise it completely.’ (Nurse other/Female/50-59 years)  ‘I think it depends what their level of understanding is in the first place. If they are very informed they might want to say ‘Look cut to the chase I don’t need to know all about kidney failure and this is a discussion that I want to have around living kidney donation’… but if you have got somebody who is a startled rabbit in the headlights, has no idea why they are even on dialysis, then maybe you need to start a little bit further back and work towards the living donor.’ (Transplant nurse or coordinator/Female/50-59 years) | **2. Tailor content to individual with respect to primary disease, kidney replacement therapy options. Home educator to agree content of session with patient prior to delivery.** | **Yes** | **2. Important for outcome** (to increase acceptability, persuasiveness, and engagement); **Consistent with guiding principles** (to increase LDKT knowledge amongst people with kidney disease and their social network, to increase an individual’s level of patient activation, to engage directly with an individual’s social support network, including potential donors)**; Uncontroversial and easy; Repeated by several participants** | **Must have** |
|  | **3. Choice of educators –professionals not patient educators**  ‘… a third party, authoritative. I mean I could probably do half of what they do in the presentation and then someone would ask me a question and I wouldn’t know. Whereas you’re talking of a professional with experience and most common questions that they are going to ask, they’ll have come across and have an answer ready.’ (Patient/Male/70-79 years/University postgraduate degree/Retired)  ‘I guess a lot of medical science actually changes over time, anyway. … I’m not sure it would be best led by somebody who was a patient, it would have to be someone who was an NHS employee.’ (Patient/Male/40-49 years/University postgraduate degree/Full-time employment) | **3. Use of professional, trained home educators** | **Yes** | **3. Important for outcome** (to increase acceptability, persuasiveness, and engagement); **Consistent with guiding principles** (to increase LDKT knowledge amongst people with kidney disease and their social network, to engage directly with an individual’s social support network, including potential donors); **Uncontroversial and easy; Repeated by several participants** | **Must have** |
|  | **4. Home visits must be undertaken by two educators – for safety and better engagement with social network:**  ‘…for the people there doing the educating it is safer to have more than one person. We will go out in pairs if we have got a very dodgy situation, so we would never go on our own if were suspicious of what was going to happen next and then you have always got that back up’ (Home dialysis nurse/Male/30-39 years)  ‘I think sometimes it is quite nice to have two. You can work off each other and they feel more involved.’ (Transplant nurse or coordinator/Female/50-59 years) | **4. Home visits to be undertaken by two home educators** | **Yes** | **4. Important for outcome** (to increase acceptability, persuasiveness, and engagement); **Consistent with guiding principles** (to engage directly with an individual’s social support network, including potential donors)**; Uncontroversial and easy; Repeated by several participants** | **Must have** |
