# Supplementary material for: Development of an intervention to improve access to living-donor kidney transplantation (the ASK study)
Source: PLoS One. 2021 Jun 25;16(6):e0253667. doi: 10.1371/journal.pone.0253667 (PMC8232417; doi:10.1371/journal.pone.0253667)
Supplement: S2 Table — (DOCX) [file pone.0253667.s002.docx]

**S2 Table. Final intervention as per the Template for Intervention Description and Replication (TIDieR) checklist(1)**

| Item No | Item |  |
| --- | --- | --- |
| **Brief name** | |  |
| 1 | Provide the name or a phrase that describes the intervention | Intervention to improve AccesS to Kidney transplantation – ASK intervention |
| **Why** | |  |
| 2 | Describe any rationale, theory, or goal of the elements essential to the intervention | Mixed-methods research has identified four variables which are key mediators of socioeconomic inequity in living-donor kidney transplantation: i) LDKT transplant knowledge, ii) patient activation, iii) perceived social support and iv) health literacy.  The intervention needs to address these identified barriers, and should therefore include the following components:  • informing kidney patients of the personal option for them of a living-donor kidney transplant (LDKT) tailored to an individual’s health literacy, to address the lack of knowledge, engagement and empowerment in treatment decision-making described.  • identification of and engagement with the patient’s social network to address the perceived lack of social support and consequent perceived lack of willing potential donors, and to overcome difficulties discussing donation; a ‘work-around’ approach to addressing limited health literacy and lack of patient activation.  • facilitation of conversations with potential donors; a ‘work-around’ approach to addressing lack of knowledge contributing to communication difficulties, lack of patient activation and a lack of perceived social support. |
| **What** | |  |
| 3 | Materials: Describe any physical or informational materials used in the intervention, including those provided to participants or used in intervention delivery or in training of intervention providers. Provide information on where the materials can be accessed (such as online appendix, URL) | - Standardised letter from healthcare professional to an individual’s potential donors with information on living kidney donation - Simple language information leaflet ‘Donating one of your kidneys’ - 10 short information animations on living kidney donation and living-donor transplantation   These resources are not currently accessible as being evaluated in an RCT and need to avoid contamination of controls. |
| 4 | Procedures: Describe each of the procedures, activities, and/or processes used in the intervention, including any enabling or support activities | 1. **Meeting between home educator and transplant candidate**   The home educator will contact the transplant candidate via telephone to arrange a meeting to discuss living-donor kidney transplantation and donation. They will ask the participant to bring contact details of their close family and friends to the meeting. The transplant candidate will meet with a home educator to discuss living-donor kidney transplantation and living kidney donation. The first animation video will be shown presenting an overview of living donor kidney transplantation. Links to the other videos will be provided to the participant. The individual will be provided with a copy of the simple language information leaflet ‘Donating one of your kidneys’. The home educator will ascertain the individual’s close relationships with relatives and friends, and the estimated age, sex, and health of these individuals. This information will be recorded and illustrated in sociogram family tree. They will discuss the initial suitability of potential donors.   1. **Standardized letter from healthcare professional to patient’s potential donors, plus simple language information leaflet ‘Donating one of your kidneys’**   The home educator will agree with the individual to which family members and close friends the standardised letter and simple language information leaflet on living donation will be posted. The patient will then address envelopes for posting to selected family members and close friends. Postage will be paid. Alternatively the patient can take home the envelopes and address these at home, or they can distribute to family and friends.   1. **Home education and engagement preparation**   The home educator will discuss the home education and engagement visit. The content of the home visit will be agreed with the participant. The proposed content is:  **Kidney disease:** *Introduction to healthy kidneys; Introduction to kidney disease - specific to the participant; The psychosocial consequences of a kidney disease and dialysis*  **Dialysis:** *The various forms of dialysis; Morbidity and mortality associated with dialysis; The advantages and disadvantages of dialysis compared to kidney transplantation*  **Transplantation:** *Introduction to transplantation; The various programs of donation and transplantation (Deceased-Donor Kidney Transplant (DDKT) and LDKT); The number of DDKT and LDKT performed nationally and locally; The differences in ethnicity regarding access to LDKT; The differences in graft survival between DDKT and LDKT*  **LDKT:** *Overview of LDKT showing animations; Additional advantages and disadvantages of LDKT; The risks and psychosocial aspects associated with donor nephrectomy; The kidney donation operation; The personal, emotional and financial aspects of LDKT for the recipient; Lifestyle after kidney donation; Common issues after kidney donation*  **Open discussion:** *Whether present individuals have any questions about kidney donation?; How do I let people know I am interested in donating?*  The home educator will agree: i) which friends and family members will be invited to the home visit, ii) where the visit will take place (home preferred location but participants may ask for an alternative location such as a community space, a hospital meeting room) iii) a number of possible dates for the home visit in the next month. If necessary the home visit can be conducted over a virtual platform and/or family and friends could join the meeting virtually.   1. **Home visit**   The home visit will be undertaken by two home educators who will deliver the education and engagement package as outlined above. The information animations will be shown and copies of the simple language information leaflet made available. The home visit can take place at a time that best suits the individual and family, including evenings and weekends.   1. **Follow up**   Within 2 weeks of the home visit the home educator will contact the patient to ask if there are any questions and if further information or support is required. If requested, a second home visit covering all or part of the content of the first visit can be delivered. |
| **Who provided** | |  |
| 5 | For each category of intervention provider (such as psychologist, nursing assistant), describe their expertise, background, and any specific training given | Intervention to be delivered by dedicated and trained home educators. Expertise in communication and education, healthcare background and knowledge of renal disease preferable. Specific training given regarding role, required knowledge, skills and tasks to be undertaken. |
| **How** | |  |
| 6 | Describe the modes of delivery (such as face-to-face or by some other mechanism, such as internet or telephone) of the intervention and whether it was provided individually or in a group | Patient participants: Patient participants will initially meet with a home educator in a face-to-face meeting, but this can be undertaken over the telephone or video-conferencing software if preferred or if required due to Covid-19 restrictions. The home visit will be undertaken by two home educators and will be a group face-to-face meeting with the transplant candidate and family. A face-to-face meeting is preferred but if required due Covid-19 restrictions it can be undertaken using video-conferencing software.  Family/friends/potential donors: Family and friends will be individually contacted by letter with an enclosed information leaflet. Those who attend the home visit will have a group face-to-face meeting, unless due to Covid-19 restrictions or individual preference the home education is delivered over video-conferencing software. |
| **Where** | |  |
| 7 | Describe the type(s) of location(s) where the intervention occurred, including any necessary infrastructure or relevant features | The transplant candidate will have their first meeting with a home educator in a place of their choosing – home or hospital. If required due to Covid-19 restrictions or the individual’s preference it can be undertaken over the telephone or via a video-conferencing tool.  The home education visit should be delivered in the transplant candidate’s home, however, an alternative private location could be considered at the individual’s request e.g. a relative’s home, a faith space. If required due to Covid-19 restrictions or the individual’s preference it can be undertaken over the phone or via a video-conferencing tool. |
| **When and How Much** | |  |
| 8 | Describe the number of times the intervention was delivered and over what period of time including the number of sessions, their schedule, and their duration, intensity, or dose | The developed intervention is due to be evaluated in a feasibility trial. The intervention as outlined is to be delivered once per transplant candidate. If requested, a second home visit covering all or part of the content of the first visit can be delivered. The home visit duration will be flexible – between 1-3 hours as required. |
| **Tailoring** | |  |
| 9 | If the intervention was planned to be personalised, titrated or adapted, then describe what, why, when, and how | The content of the home education visit will be tailored for the individual patient recipient with respect to their primary renal disease, their current stage of kidney disease, their individual treatment options. |
| **Modifications** | |  |
| 10 | If the intervention was modified during the course of the study, describe the changes (what, why, when, and how) | Currently N/A – due to be evaluated in a feasibility trial |
| **How well** | |  |
| 11 | Planned: If intervention adherence or fidelity was assessed, describe how and by whom, and if any strategies were used to maintain or improve fidelity, describe them | Currently N/A – due to be evaluated in a feasibility trial |
| 12 | Actual: If intervention adherence or fidelity was assessed, describe the extent to which the intervention was delivered as planned | Currently N/A – due to be evaluated in a feasibility trial |

**References**

1. Hoffmann T, Glasziou P, Boutron I, Milne R, Perera R, Moher D, et al. Better reporting of interventions: template for intervention description and replication (TIDieR) checklist and guide. BMJ. 2014;348:g1687.
