# Supplementary material for: Development of an intervention to improve access to living-donor kidney transplantation (the ASK study)
Source: PLoS One. 2021 Jun 25;16(6):e0253667. doi: 10.1371/journal.pone.0253667 (PMC8232417; doi:10.1371/journal.pone.0253667)
Supplement: S1 File — (DOCX) [file pone.0253667.s003.docx]

**S1 File. Example topic guide – for patient participant interviews**

| **Topic** | **Questions** |
| --- | --- |
| Communication on LDKT | Have you talked to your family and close friends about the possibility of having a kidney transplant? Was/Is this difficult? If yes, what makes it difficult? Did anything make it easier? |
|  | Did you ever directly ask somebody to give you a kidney? What happened? |
| Attitudes towards obtaining and providing information | Did/Do you feel you have enough information on kidney transplantations and kidney donation? |
|  | Did/Do you feel happy you understood/understand everything? |
|  | Are you happy to ask your doctor or kidney nurse questions if you have them? Is this easy to do? |
| Norwegian approach | In Norway the kidney doctor asks the person with kidney disease about who is in their family and any close friends. If the patient agrees, the doctor then contacts these family members and friends asking them to think about donating a kidney. They are invited to come to the hospital to talk about kidney donation.  What do you think of this approach? |
|  | Does anything appeal to you about this? Does anything worry you about this? |
|  | Discuss wording/content of letter and living donation information sheet. Review drafted resources. |
| Home-based educational intervention | In the Netherlands and in parts of America, nurses or psychologists visit people with kidney disease and their families at their homes. People are encouraged to invite any family members and friends who don’t live at home to come to the meeting. The nurses/psychologists talk to everyone about kidney disease, transplants and kidney donation, they help to start conversations about possible kidney donation, and can answer any questions people have face to face. They can visit once or twice, and leave information sheets, DVDs, website links etc.  What do you think of this approach? |
|  | What would you like to discuss during such a meeting? |
|  | Do you think that people in your family would be open to this? |
|  | Can you think of people who might not want something like this? |
|  | Do you think other places (e.g. café, church) would be alternatives to going into someone’s home? |
|  | The nurses and psychologists usually take information sheets and can provide DVDs and weblinks. Do you think this is helpful? |
|  | Discuss animation content. |
| Transplant Candidate Advocates (TCAs) | People tell us it can be very difficult to talk to family and friends about possible kidney donation.  In some parts of America, doctors ask people with kidney disease to pick a family member or friend who receives training on living-donor kidney transplantation, and in how to start conversations about kidney donation. This person is given information leaflets and is trained as an advocate: someone willing to speak to other friends/family about donation on the patient’s behalf. They can also go to clinic appointments with the patient and ask questions and find out information.  What do you think of this approach? |
|  | Can you think of anyone you might ask to do this? |
|  | Do you think that people in your community would be open for this intervention? |
| Other suggestions | Is there another approach you think might be helpful? |
| Opt-out  legislation | In May 2020 England will move to an ‘opt-out’ law for organ donation after death. Were you aware of this? What do you think about this? |
|  | What impact do you think the law change will have? How do you think the change in the law will affect you? Probe if participant expects law change to increase number of organs available for transplant. |
|  | Will the change in the law affect any of the decisions you’ve made about transplants? Probe as to whether it would change willingness to accept a living donor kidney transplant. |
|  | Do you think the change in law should be highlighted in a media campaign, for example on TV? Investigate participant views on content of adverts. |
|  | Do you think a media campaign should focus on donation after death or should it include living donation? |
