# Supplementary material for: Development of an intervention to improve access to living-donor kidney transplantation (the ASK study)
Source: PLoS One. 2021 Jun 25;16(6):e0253667. doi: 10.1371/journal.pone.0253667 (PMC8232417; doi:10.1371/journal.pone.0253667)
Supplement: S2 File — (DOCX) [file pone.0253667.s004.docx]

**S2 File. MoSCoW (Must have, Should have, Could have, Would like) criteria (1, 2)**

**MoSCoW** is a prioritisation model that can be usefully employed to prioritise the content of interventions (1, 2):

**Must have** – the modification must be made for the intervention to be effective, acceptable and feasible;

**Should have** – the modification should be made if possible as it may affect effectiveness but is less critical than a Must have;

**Could have** – this modification would be useful but may be less critical to effecting change than a Should have, and may only be implemented if time and resources allow; and

**Would like** – this modification is not needed to support change but could be useful if time and resources allow(2).

**References**

1. Bradbury K, Watts S, Arden-Close E, Yardley L, Lewith G. Developing digital interventions: a methodological guide. Evid Based Complement Alternat Med. 2014;2014:561320.

2. Bradbury K, Morton K, Band R, van Woezik A, Grist R, McManus R, et al. Using the Person-Based Approach to optimise a digital intervention for the management of hypertension. PLoS One. 2018;13(5):e0196868.
